# Dynamic behaviour of human neuroepithelial cells in the developing forebrain

**DOI:** 10.1038/ncomms14167

**Published:** 2017-01-31

**Authors:** Lakshmi Subramanian, Marina Bershteyn, Mercedes F. Paredes, Arnold R. Kriegstein

**Affiliations:** 1 Eli and Edythe Broad Center of Regeneration Medicine and Stem Cell Research, University of California San Francisco, 35 Medical Center Way, San Francisco, California 94143-0525, USA; 2 Department of Neurology, University of California, San Francisco, California 94143, USA; 3 Present address: Neurona Therapeutics, 650 Gateway Blvd Ste 125, South San Francisco, California 94080, USA

## Abstract

To understand how diverse progenitor cells contribute to human neocortex development, we examined forebrain progenitor behaviour using timelapse imaging. Here we find that cell cycle dynamics of human neuroepithelial (NE) cells differ from radial glial (RG) cells in both primary tissue and in stem cell-derived organoids. NE cells undergoing proliferative, symmetric divisions retract their basal processes, and both daughter cells regrow a new process following cytokinesis. The mitotic retraction of the basal process is recapitulated by NE cells in cerebral organoids generated from human-induced pluripotent stem cells. In contrast, RG cells undergoing vertical cleavage retain their basal fibres throughout mitosis, both in primary tissue and in older organoids. Our findings highlight developmentally regulated changes in mitotic behaviour that may relate to the role of RG cells to provide a stable scaffold for neuronal migration, and suggest that the transition in mitotic dynamics can be studied in organoid models.

## Abstract (web summary)

The dynamics of progenitor cells in human neocortex development has not been studied directly. Here, the authors timelapse image human neuroepithelial (NE) and radial glial (RG) cells in embryonic brain slices and find properties of NE cells and RG that are mimicked in cerebral organoids.

The expansion of the human cerebral cortex during evolution is thought to be the result of an increase in the number and diversity of progenitor cells that give rise to cortical neurons^1^,^2^. Many recent studies have focused on identifying and characterizing the behaviours of the progenitors that either directly and/or indirectly generate these neurons^3^,^4^,^5^,^6^. The radial glial (RG) cell has been identified as the primary progenitor cell in the mammalian cortex that can both self-renew and generate neurons^7^,^8^. More recent studies have identified several other progenitor subtypes, including intermediate progenitor cells (IPC)^9^,^10^,^11^,^12^,^13^ and outer RG^5^,^14^,^15^ that are all generated by RG cells and contribute to an overall increase in neuronal number. According to the radial unit hypothesis of cortical development, these diverse progenitor cell types arise from a parent population of neuroepithelial (NE) cells that are the founder cells of the nervous system^16^. As part of the neural plate and the early neural tube, NE cells contribute to the structure and shape of the developing nervous system. When the neural tube regionalizes in response to morphogens and signalling molecules, the anterior end expands to generate the telencephalon. NE cells contribute to this expansion through proliferation.

NE cells were first described in 1889 by His^17^ in the neural tube of the human embryo. This was also one of the earliest descriptions of the characteristic localization of mitotic NE cells to the interior or luminal surface of the neural tube. Later studies by Sauer^18^ in the neural tube of pig and chick embryos, confirmed that mitosis at the lumen surface was a characteristic feature of the vertebrate neuroepithelium and established the apico-basal polarity of NE cells with the apical side exposed to the lumen and the basal side attached to the basal lamina. This study also first introduced the model for interkinetic nuclear migration (INM), in which the nucleus of the parent cell translocates to the apical side during mitosis and the daughter nuclei migrate away after mitosis^19^. Many subsequent studies, using modern molecular characterization and immuno-histochemical localization in a variety of species including zebrafish, chick and mouse, have confirmed the essential characteristics of NE cells: their apico-basal polarity, INM, and apical mitosis^20^,^21^,^22^.

Early studies^18^ suggested that mitotic NE cells round up at the lumen, retract their processes before division and regenerate a basal fibre following mitosis. Later electron microscopic and other observations of the ultra-structure of mitotic cells appeared to confirm these observations^23^,^24^,^25^,^26^,^27^,^28^. More recent studies on proliferating RG using live-imaging techniques, however, clearly showed that RG cells retain their basal processes during mitosis^8^,^29^. Other reports described a basal process on mitotic cells in some instances but did not find them to be consistently present^30^. It has been suggested that the basal process splits during division, a process that could allow the symmetrical distribution of apical domains in proliferative progenitors^20^,^31^. Each of these observations was made in progenitor cells from different species and at different ages. Furthermore, some were based on genetic labelling of precursors already committed to the neuronal lineage, thereby bypassing the parent progenitor population^32^,^33^,^34^,^35^. Finally, studies on the early telencephalic neuroepithelium in primates and humans have been limited to descriptions of stained cells and in fixed samples^28^,^36^,^37^,^38^. The conflicting literature underscores the gaps that remain in our understanding of the dynamics of the proliferative divisions during early fetal development.

NE and RG cells share many morphological and molecular features, but they differ in their proportion of proliferative, symmetric divisions that expand the neuroepithelium and other divisions that serve to increase cellular diversity. These differences are of particular interest in understanding the development of the human cerebral cortex, which has undergone an evolutionary expansion in the number of NE progenitors as well as an increase in progenitor diversity. In an effort to better understand the cellular dynamics of proliferative NE cell divisions in humans, we undertook a systematic analysis of the early human telencephalic neuroepithelium. Using live-imaging techniques to follow GFP labelled proliferating cells, we show that human NE cells undergo self-renewing, symmetric proliferative cell divisions that are clearly distinct from the cell division patterns observed in RG cells. Unlike RG cells, mitotic NE cells retract their basal fibres before division. This distinctive mitotic behaviour can be recapitulated in cerebral organoids generated from induced pluripotent stem (iPS) cells. We show that proliferating cells in younger organoids have the characteristic mitotic pattern of NE cells, while proliferating cells in older organoids display RG cell dynamics. Taken together our observations suggest a symmetric dynamic pattern of cell division for NE cells that serves to expand the progenitor cell pool before neurogenesis, with a shift to a more anchored polarized pattern of division characteristic of RG cells, possibly related to the asymmetric fates of their daughter cells, and/or their additional role in guiding neuronal migration.

## Results

### Regional identity and location of mitotic NE cells

**Figure 1:**
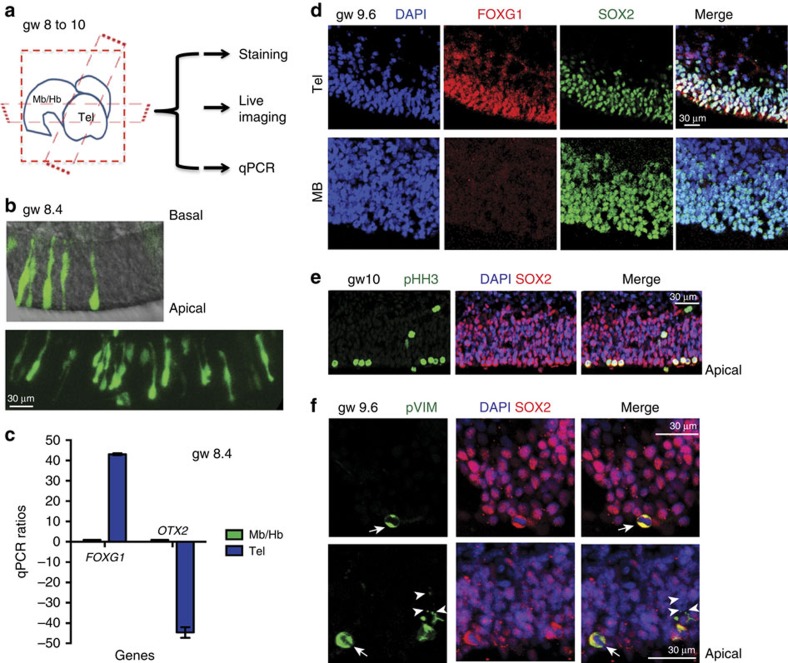
Regional identity and location of mitotic NE cells. (**a**) First trimester human brain tissue was separated into probable anatomical regions: Telencephalon (Tel), Mid-brain or Hindbrain (Mb/Hb). Each region was sectioned in one of the three planes to expose the neuroepithelium (NE). The slices were then further processed for live imaging, immuno-staining and qPCR analysis. (**b**) Appearance of the telencephalic NE following viral GFP labelling. (**c**) qPCR analysis of a GW8.4 sample. qPCR ratios are the relative expression levels determined using the Pfaffl method^57^,^58^, taking into account primer efficiency. *FOXG1* expression is several fold higher in a telencephalic slice than in a mid-brain slice from the same sample. In contrast, *OTX2* expression is decreased several fold in the telencephalic slice. (**d**) Immunofluorescence data showing nuclear expression (co-localization with DAPI in single plane confocal images) of FOXG1 in the SOX2-expressing cells of the telencephalon. SOX2-expressing cells of the mid-brain do not express FOXG1. (**e**,**f**) Mitotic cells in the telencephalic neuroepithelium. (**e**) phospho-histone H3 (pHH3) positive mitotic cells express SOX2 and are predominantly localized to the apical surface of the neuro-epithelium at gw10. (**f**) Mitotic intermediate filament marker phospho-vimentin (pVIM) reveals the morphology of the mitotic cells. Cells that have no basal process (white arrows) as well as cells with a thin basal process (white arrowheads) are both present in the neuroepithelium. Scale bars, 30 μm.

To investigate the properties of a proliferating stem cell pool in the human telencephalon, we undertook a systematic analysis of the telencephalic neuroepithelium during the first trimester. Neural tissue samples between gestational week (GW) 8 and GW10 were visually separated into broad regions (spinal cord, mid-/hind- brain and telencephalon). Each regional fragment was sectioned in one of three orthogonal planes to expose the neuroepithelium (Fig. 1b). The resultant organotypic slices were distributed for live imaging, RNA extraction for gene expression analysis and fixed for immunofluorescence staining.

We used immunostaining, gene expression analyses or both to confirm the telencephalic and regional identity of each tissue sample. Gene expression was analyzed by quantitative PCR following reverse transcription (RT-qPCR) and it showed that forebrain tissue expressed several fold higher levels of transcript for the telencephalic marker *FOXG1* as well as several fold lower levels of *OTX2* transcript when compared with the mid-brain (Fig. 1c). In the forebrain, FOXG1 expression was seen in the nuclei of cells expressing the stem cell marker SOX2 (Fig. 1d). At the same time, FOXG1 expression was absent from the SOX2-expressing cells in the mid-brain. RT-qPCR analyses showed a nearly twofold increase in the transcript for dorsal marker *PAX6* (ref. 39) in the telencephalon when compared with the mid-brain (Supplementary Fig. 1a). This was validated by immunostaining data showing PAX6 staining in the SOX2-expressing cells of the telencephalon but not in the SOX2-expressing cells of the mid-brain (Supplementary Fig. 1b). The SOX2-expressing cells in the telencephalon also expressed the dorsal marker EMX2 (ref. 40) (Supplementary Fig. 1c). Further, immunostaining with mitotic markers, phospho-histone H3 (pH-H3) and phospho-vimentin (pVIM), confirmed the apical location of dividing cells (Fig. 1e,f) consistent with NE organization. Together with the combination of FOXG1, PAX6 and EMX2 expression, this confirms the dorsal telencephalic NE identity of the tissue used in subsequent studies.

pVIM staining also revealed a range of different cell morphologies in the mitotic cells of the cortical neuroepithelium (Fig. 1f). Some pVIM-positive cells (white arrows in Fig. 1f) at the apical surface appeared to be clearly rounded with no basal fibres. Other apically located cells had basal fibres (white arrowheads in Fig. 1f), although apical processes were absent in all the cells.

### NE cells undergo proliferative divisions

The diverse range of cell morphologies in pVIM-positive cells suggested that they might be very dynamic in their behaviour during mitosis. To examine the behaviour of NE cells, we labelled individual cells in thick organotypic slices by infecting them with a GFP expressing adenovirus. We observed labelled NE cells within 24 h of infection (Fig. 1b). We followed the behaviour of the cells as they divided using a real-time imaging system that allowed us to capture images at a frame rate of once every 4–6 min over 1–5 days.

We observed that the early telencephalic neuroepithelium was highly proliferative, and that individual cells were in a very dynamic state throughout the imaging period, either undergoing INM or mitosis (Supplementary Movie 1). We determined the length of the cell cycle for NE cells by following labelled mitotic cells through more than one cell cycle and defined the cell cycle length as the interval between the initiations of two consecutive cytokinetic events. We obtained a mean cell cycle time of 36±7 h (*n*=10 cells). The shortest cell cycle time observed was 23 h while the longest was 46 h.

**Figure 2:**
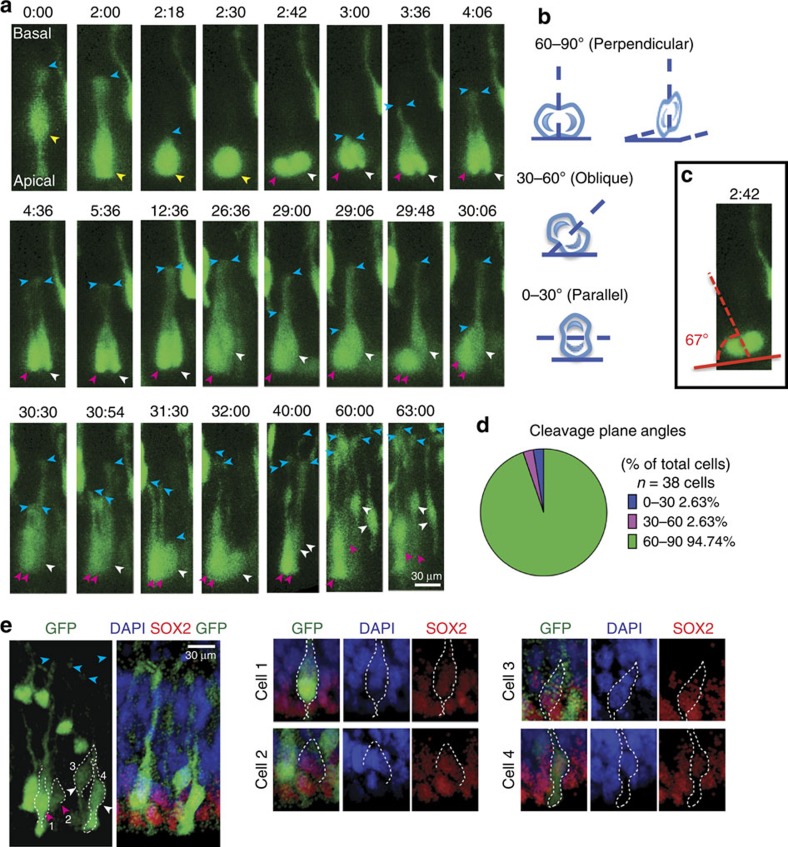
NE cells undergo proliferative divisions. (**a**) Still frames of one NE cell show the cell undergoing multiple rounds of cell division: The spindle shaped parent neuro-epithelial cell translocates its nucleus (yellow arrowhead) to the apical surface where it undergoes mitosis to generate two daughter cells (magenta and white arrowheads). The nuclei of both daughter cells undergo INM and translocate to the apical surface for a second round of mitosis. The basal processes (blue arrowheads) of the dividing parent and daughter cells show retraction and re-growth. (**b**) Schematic representation showing the measurements of the cleavage plane angle at the apical surface: cell divisions with a 60–90° cleavage plane are perpendicular, 30–60° cleavage plane angles are oblique and 0–30° cleavage plane angles are parallel. (**c**) The cleavage plane of the first cell division is at a 67° angle to the apical surface and is therefore perpendicular. (**d**) Quantification of the cleavage plane angles of 38 NE cells shows that over 94% of the divisions are perpendicular. (**e**) Post-imaging staining shows that all four daughter cells (magenta and white arrowheads from (**a**)) continue to express the stem cell marker SOX2. Scale bars, 30 μm.

**Figure 3:**
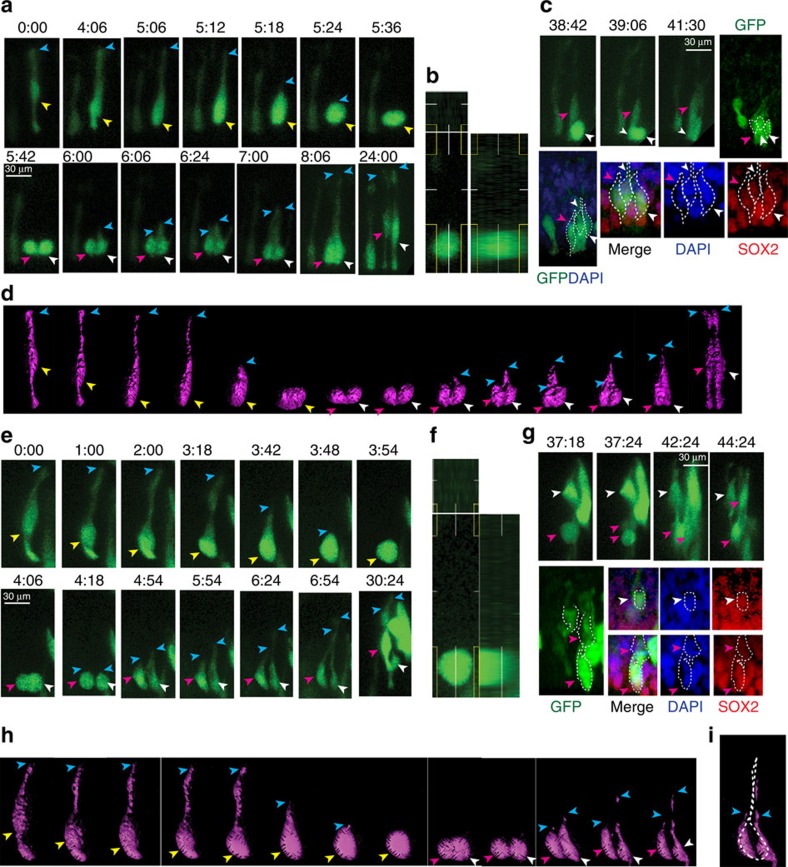
NE cells retract their basal process during mitosis. Still frames of two different NE cells during mitosis highlighting changes in the basal process (**a**,**e**): the parent cells (yellow arrowheads) translocate their nucleus to the apical surface and begin to round up. The basal process shortens simultaneously (blue arrowheads) and begins to retract towards the nucleus. At mitosis, only the round nucleus is visible. Following mitosis, each daughter cell (magenta and white arrowheads) independently re-grows its basal process (blue arrowheads). (**b**,**f**) Analysis of the *yz* and *xz* confocal projection frames at mitosis shows complete absence of the basal process in both cell divisions. (**c**,**g**) The daughter cells (magenta and white arrowheads) undergo additional proliferation and continue to express SOX2 as shown by post-timelapse immuno-staining. (**d**,**h**) 3D surface reconstructions of cells in (**a**,**e**) clearly demonstrate the retraction of the basal process in NE cells during mitosis and the subsequent re-growth of the fibre in daughter cells (**i**) Super-imposing the outline of the parent cell (white dotted lines) on the 3D reconstructed daughter cells shows that the basal processes of the daughter cells (blue arrowheads) are distinct from the basal process of the parent cell. Scale bars, 30 μm.

To determine whether the NE cells were undergoing divisions to generate other NE daughters, we used samples that were less densely labelled and analysed the characteristic features of NE cell division in 40 cells from three independent tissue samples. In 10 of these divisions, at least one of the daughter cells underwent an additional round of division (Figs 2a, 3c,g), while in two cases, both daughter cells went through an additional round of division to generate four daughters (Supplementary Movie 2 and Supplementary Fig. 2a). To determine the post mitotic fate of the NE and daughter cells, we stained the imaged samples for markers of proliferation and differentiation. All the daughter cells that we examined (30/30) continued to express the stem cell marker, SOX2 (Fig. 2e), and none of them expressed the IPC marker, TBR2 (ref. 41; Supplementary Fig. 2), or the neuronal marker TUJ1, suggesting that they did not differentiate into either of these cell types. We also observed that at these early ages, the neuroepithelium uniformly expressed SOX2 but did not express any markers of differentiation like TBR2 (IPCs) or CTIP2 (neurons^42^,^43^; Supplementary Fig. 2), suggesting that this stage was before the start of neurogenesis.

The orientation of the mitotic spindle in proliferative epithelial cells has been suggested to predict whether cell division is symmetric (giving rise to identical daughter cells) or asymmetric (giving rise to different daughter cells)^44^. In ventricular RG (vRG), symmetric divisions are thought to occur when the mitotic spindle is parallel to the apical surface and the plane of cytokinesis is perpendicular (Fig. 2b). Most proliferative divisions in vRG cells would fall into this category although asymmetric divisions can also have this cleavage orientation^45^. Alternatively, asymmetric divisions that renew the parent cell while producing a differentiated daughter cell (IPC, oRG or neuron) are thought to be more likely when the cleavage furrow is oblique or parallel to the apical surface (Fig. 2b). This difference in cell fate as a function of the cleavage plane orientation has been proposed to result from differences in the distribution of the apical domains to daughter cells during mitosis^20^. A predominantly vertical cleavage plane of NE cell divisions would be most consistent with proliferative symmetric divisions. We measured the angle of the observed cleavage plane to the apical surface in 38 mitotic NE cells and 94% (36/38) of the cells showed a perpendicular cleavage plane (Fig. 2d). Thus, a vast majority of the mitotic NE cells have a cleavage angle consistent with symmetric division and self-renewal.

### NE cells retract their basal process during mitosis

A characteristic feature of vRG divisions in the telencephalon is that they continue to maintain contact with the basal lamina through a retained basal fibre throughout the cell cycle. NE cells are molecularly similar to vRG cells to which they are closely related, but it is not clear if they show the same property of basal fibre retention. Some studies have suggested that mitotic NE cells may retract their basal fibre during anaphase and telophase, while others suggest that the fibre may be retained^18^,^23^,^28^,^30^. Several models of how basal fibre retention may occur in early proliferative divisions have been proposed, including splitting of the basal fibre^22^,^31^.

We therefore examined the behaviour of GFP labelled NE cells in organotypic slices as described above. We examined the images collected during timelapse imaging of these cells, and performed a frame by frame analysis of the morphology of individual NE cells before, during, and after mitosis. In particular, we closely examined the behaviour of the basal process throughout the cell cycle. We observed that 100% (40/40) of NE cells retracted their basal process during mitosis (Supplementary Movies 1 and 2, Supplementary Figs 2a,3a,b,d–h). Such a retraction was never observed in any non-mitotic GFP-labelled cells. Typically, a mitotic NE cell translocated its nucleus to the apical surface, and coincident with rounding up, the basal process became thinner and slowly shortened in length, pulling away from the basal surface (blue arrowheads in Fig. 3a,d). In some of the cells (29.6%; 8/27 cells from three independent samples), the retraction of the basal process began before the nucleus reaching the apical surface. As mitosis proceeded, the basal process of the NE cell was fully retracted and cytokinesis occurred in the rounded parent cell. The time interval between apical nuclear translocation and cytokinesis was variable with cells dividing almost instantaneously or after as long as 5.5 h (mean 91.2 min; *n*=27 cells). However, once the cell began to retract its basal process, cytokinesis occurred fairly rapidly, always within an hour (mean 37.3 min; *n*=27 cells). The two daughter cells thus generated briefly appeared round, but then rapidly and independently generated their own basal processes that extended to and soon reached the basal lamina. Typically, the time interval between cytokinesis and the beginning of basal process re-growth was <30 min (*n*=27 cells; mean 11.5 min). The daughter cell nuclei then translocated away from the ventricular surface. In time (2–25 h), the daughter cells regained the typical spindle shaped appearance of NE cells with an apical and basal process spanning the width of the neuroepithelium (Fig. 3a; time point 24:00).

Our rapid frame rate during live imaging of the neuroepithelium allowed us to examine the morphological changes of the basal fibre in detail. We followed each dividing NE cell over several frames during rounding-up and basal retraction. In every cell we observed, retraction of the basal fibre was a unidirectional and progressive event; that is, once retraction began, the basal process shortened in each successive frame until it disappeared in the rounded-up cell before cytokinesis. This direct observation of fibre retraction rules out fibre thinning as a cause of apparent NE fibre retraction. Further evidence for retraction of the basal process during mitosis is provided by our analysis of the three-dimensional (3D) confocal projection data for each cell. We examined the *xy*, *yz* and *xz* projection planes for each individual mitotic cell (*n*=40 cells). No basal fibre was observed in any of these projection planes for mitotic cells at this stage (Fig. 3b,f). We generated 3D surface reconstructions of the mitotic cells (Fig. 3d,h) that clearly demonstrate the retraction of the basal process during mitosis and the subsequent re-growth of the fibre in daughter cells.

The behaviour of both daughter cells following mitosis further supports a model in which the basal fibres retract and regrow during NE cell divisions. We visualized the re-growth of the basal process in each daughter cell and observed a growth cone at the extending tip as well as sprouting and branching behaviours (Supplementary Movie 2; time point 0:16). These growth cones would be absent if this were simply ‘inflation' of an already existing basal fibre. Further, the position of the newly regrown basal process in each daughter cell is often distinct from the original position of the retracting basal process in the parent cell before division (Fig. 3i). Taken together, the data indicate that, unlike in RG cells where the basal fibre of the parent cell is inherited by the self-renewed RG daughter cell, both daughters of a dividing NE cell grow new basal fibres.

### RG proliferation in the early neuroepithelium

Although NE-type cell divisions as described above were the predominant cell division pattern in the early human telencephalon (GW 8–10), we noticed that some of our older first trimester organotypic slices (GW 9–10) contained cells that showed mitotic behaviours with basal fibre retention similar to RG cells that had been imaged in more mature tissue. We occasionally observed NE stem cell divisions and RG-like cell divisions in the same slice (*n*=12 cells in five slices) (Supplementary Movie 3). These slices, therefore, provided us with an opportunity to compare and contrast the behaviour of the earliest RG cells with that of NE stem cells. We observed mitotic behaviour that resembled the pattern described for older RG^3^. The somata of the presumed RG cells underwent INM within the ventricular zone, translocated to the apical surface, and swelled during mitosis. In contrast to NE type divisions, however, the presumptive RG cells continued to maintain contact with the basal lamina via the basal fibre throughout M-phase. During, M-phase, the basal fibre appeared to thin out and sometimes became nearly undetectable except for intermittent varicosities, as has been previously described for RG cell division^8^,^29^. Following mitosis, the fibre was retained by one of the daughter cells, thickened and became detectable again along its whole length. Meanwhile, the other daughter generated its own basal fibre, using the basal fibre of the sister cell as a guide (Supplementary Movie 3 and Supplementary Fig. 4a).

**Figure 4:**
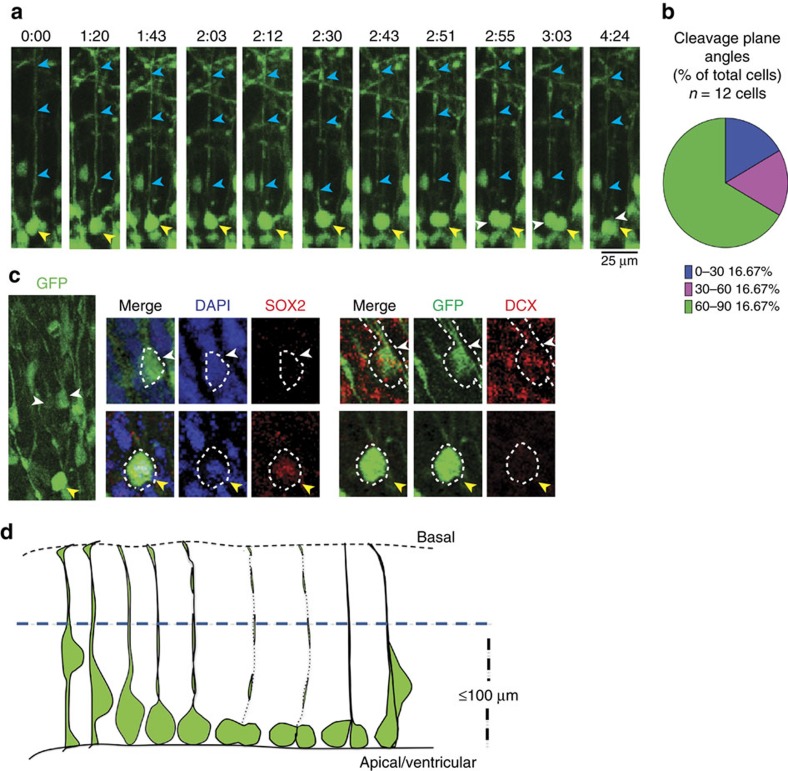
RG proliferation in the early neuroepithelium. Still frames of a RG cell showing the retention of the basal process during mitosis (**a**): The nucleus (yellow arrowhead) of the parent RG cell is seen in the ventricular zone while a long thin process extends basally (blue arrowheads) to the pial surface. As the cell prepares for mitosis, the nucleus moves closer to the apical surface. At mitosis, the basal process shows significant thinning and is almost invisible except for the varicosities present along its length but it does not shorten. Following mitosis, the basal process thickens again and is more easily seen. (**b**) Quantification of the cleavage plane angles of 12 RG cells shows most (66.6%) of these divisions are still perpendicular to the apical surface while 16.6% of the divisions are oblique and 16.6% of the divisions are parallel to the ventricular surface. (**c**) Post-timelapse immuno-staining of daughter cells shows a SOX2 positive daughter (yellow arrowhead) and a differentiated daughter expressing DCX (white arrowhead). (**d**) Model demonstrating the mitotic behaviour of RG cells: The parent cell nucleus translocates to the apical surface and rounds-up for mitosis. The basal process thins and varicosities appear, but the length of the process does not change. Following mitosis, the basal process thickens and can be seen associated with the daughter cells. The parent and daughter cells show INM within a ventricular zone that is ∼100 μm from the apical surface. Scale bars, 25 μm.

We were able to identify 12 cells that showed this characteristic ‘radial glial' mitotic pattern in our first trimester samples. We examined in detail the imaging frames generated for each of these cells and analysed the cleavage plane angles of parent RG cells with respect to the apical surface. Unlike, NE cell divisions, 68% of the RG-like cell divisions showed a perpendicular cleavage plane. This number is consistent with reports of the spindle orientations and cleavage plane angles of RG cells^46^. Thirty-two per cent of the divisions were in an oblique or parallel plane, suggesting that these cells might be generating other progenitor cell types or differentiated neurons. We further explored the possibility of asymmetric division in these presumed RG cells through an analysis of post-imaging cell fate markers (Fig. 4c). Four out of 12 RG-like daughter cells that we examined no longer expressed SOX2 but instead expressed markers of differentiation-like DCX (Fig. 4c).

We observed that cells showing RG-like mitosis had longer basal processes than the NE-like mitotic cells (Supplementary Fig. 1). Typically, cells showing NE cell type divisions had an overall mean length of 70.95±10 μm (*n*=38 cells), while RG-like dividing cells had a mean length of 156.6±32 μm (*n*=12 cells) with basal processes that turned to run obliquely through the marginal zone. The nuclei of RG-like cells, however, did not migrate throughout the entire cell fibre but only travelled a mean of 58.82±19 μm from the apical surface, similar to the 51.26+13 μm traversed by the nuclei of NE cells, establishing a *de facto* ‘ventricular zone' that corresponds roughly to the width of the early neuroepithelium.

### Modelling early mitotic behaviours in cerebral organoids

Three-dimensional cerebral organoids are emerging as a complementary model system to study features of early human cortical development and disease^47^. However, the extent to which dynamic properties of neural stem and progenitor cells are cell-autonomous and can be ascertained *in vitro* has been unclear. We applied a published method^47^ to generate cerebral organoids from wild-type (WT) iPSCs derived from three healthy individuals (*N*=3) to determine whether basal process dynamics of dividing NE cells and vRG cells faithfully recapitulate the behaviours we observed in primary tissue. After 5 or 10 weeks of differentiation, we fixed 10–20 organoids from each individual and performed serial sections of entire organoids followed by immunostaining (Supplementary Fig. 4). This was repeated in 4–6 independent differentiation experiments for each of the lines (Supplementary Fig. 4b). At 5 weeks, the organoids consisted of multiple pseudo-stratified VZ-like progenitor regions (Supplementary Fig. 4c). Dorsal telencephalic patterning was verified by expression of progenitor marker PAX6 (ref. 39) in the VZ-like regions, which were surrounded by DCX-positive neurons also expressing deep layer subcortical projection neuron marker CTIP2 (refs 42, 43; Supplementary Fig. 4c). At ten weeks of differentiation, cortical organoids were characterized by the appearance of IPC marker TBR2/EOMES^41^ (Supplementary Fig. 4d).

We found that cortical lineage specification was consistent between ‘sister' organoids within a given differentiation experiment (Supplementary Fig. 4c). However, inter-experimental efficiency of cortical specification as defined by PAX6, Tbr2 and CTIP2 expression was dependent on the line, with line# 1323 being the most robust (Supplementary Fig. 4b). Although WNT and TGFβ pathways inhibitors were used to promote dorsal forebrain specification and suppress other lineages, our observations suggest that intrinsic differences between iPSC lines can contribute to inconsistent patterning, as observed previously^48^. Thus, further optimization of small molecule conditions is necessary to increase the efficiency of dorsal telenchephalon specification across individuals and lines than what is currently achieved with this protocol. Nonetheless, using the PAX6, Tbr2 and CTIP2 expression criteria, we were able to focus all subsequent analyses on the organoids that were verified to express cortical markers.

**Figure 5:**
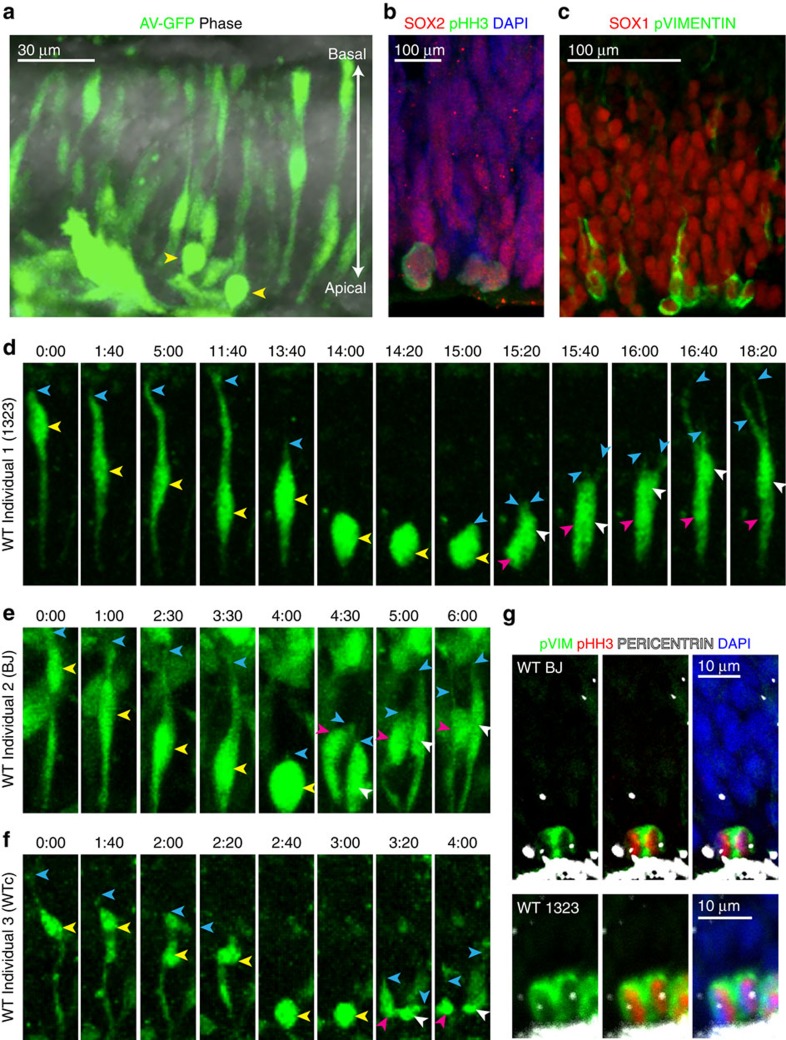
NE cells in week 5 organoids retract the basal process during mitosis. (**a**) Characteristic spindle shaped morphology of NE cells following viral GFP labelling. (**b**,**c**) Mitotic cells in 5-week-old organoids identified by pHH3 or pVIM staining localize to the apical surface and express the stem cell markers Sox1 and Sox2 (**d**–**f**) Examples of NE cell division in 5-week-old organoids from three different individuals, all showing that the basal process completely retracts and re-grows independently in the two daughters. (**g**) pVim staining in mitotic NE cells shows no evidence of a basal process at anaphase. The mitotic stage was identified by the distribution of chromatin by DAPI staining and pHH3 expression, as well as by the position of the centrioles labelled by pericentrin.

GFP-labelling of week 5 organoids revealed elongated cell morphology that is strikingly similar to primary NE cells (compare Figs 1b and 5a). Immunostaining with antibodies against apical determinants such as N-CADHERIN and PERICENTRIN revealed a clear apico-basal polarity (Supplementary Figs 4c and 5g). Mitotic cells expressing pHH3 or pVIM were always observed at the apical surface (Fig. 5b,c and Supplementary Fig. 5a,b), similar to *in vivo.*

We used timelapse imaging of GFP-labelled cells to carefully examine dynamic properties of dividing cells (*n*=10–30 from each of three WT iPSC lines) after 5 weeks of differentiation. We consistently observed characteristic INM behaviour where the parent cell nucleus moved to the apical surface and began to round up for mitosis (yellow arrowheads in Fig. 5a,d–f), while the basal fibre retracted completely into the dividing cell (blue arrowheads in Fig. 5d–f). Following cytokinesis, each daughter cell (magenta and white arrowheads) independently regrew its basal fibre (blue arrowheads in Fig. 5d-f). We closely examined the behaviour of the basal process throughout the cell cycle in 17 cells from three different WT iPSC lines in 5-week-old organoids. We observed that all 17 cells retracted their basal process during mitosis. Similar to NE cells from primary tissue, mitotic cells in the organoids translocated their nucleus to the apical surface. As the cells rounded up, the basal process slowly shortened in length and pulled away from the basal surface. In about a third of the cells (35.2%; 6/17 cells), the retraction of the basal process began before the nucleus reaching the apical surface (compared with 29.6% cells in primary NE). Cytokinesis occurred in the rounded parent cell after full retraction of the basal process. Cells typically divided between 20 min to 2 h after apical nuclear translocation (mean 51.18 min; *n*=17 cells). 88% (15/17) of cells divided within an hour of retracting their basal process (mean 39.3 min; compared with mean 37.3 min in primary NE cells). The two daughter cells regrew their basal process within 60 min (*n*=17 cells; mean 29.5 min) and the daughter cells regained their typical spindle shaped appearance within 12 h. The timing of these cellular events is broadly similar in both primary NE cells and week 5 organoid cultures. This suggests that 5-week-old organoids are a reasonably faithful model of the behaviour of early NE cells.

Furthermore, immunostaining against pVim did not reveal persisting basal processes in anaphase cells (Fig. 5g, *n*=83), whereas a small fraction of mitotic cells that were at a stage before anaphase exhibited a basal fibre (31/365, or 8.5%) (Supplementary Fig. 5a,b,d). These *in vitro* findings further support the notion that NE cells completely retract the basal fibre during division, suggesting that this is a cell autonomous property characteristic of this stage of cortical development.

**Figure 6:**
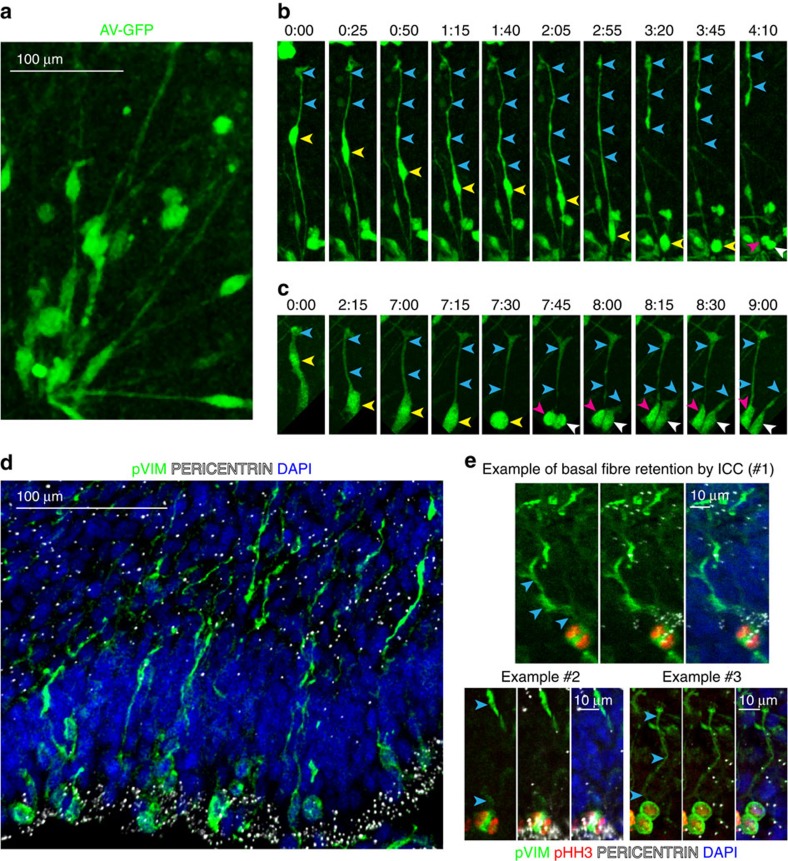
RG cells in week 10 organoids retain the basal process during mitosis. (**a**) Viral GFP labelling highlights the characteristic morphology of RG cells in week 10 organoids (**b**,**c**) Examples of RG divisions in 10 week old organoids from two different individuals showing that the basal process remains associated with one of the daughter cells. (**d**) pVIM staining of week 10 organoid at low magnification, showing many relatively long basal processes. DAPI labelling highlights the nuclear organization of the organoid while PERICENTRIN labelling highlights the apical surface. (**e**) pVIM staining in mitotic RG shows that the basal process (blue arrowheads) is retained during anaphase/telophase. The mitotic stage was identified by the distribution of chromatin in DAPI staining and pHH3 expression as well as by the position of the centrioles labelled by PERICENTRIN.

Next, we examined cell morphology and mitotic behaviour of RG-like cells in organoids at 10 weeks of differentiation. GFP-labelled cells in VZ-like regions appeared to be longer, with thinner apical and basal processes (Fig. 6a), similar to primary RG. Live-imaging studies revealed dividing RG-like cells undergoing INM (Fig. 6b,c), similar to the earlier time point. However, in contrast to the completely retracting basal fibres of dividing NE-like cells in 5-week organoids, we observed examples of dividing cells with persisting basal fibre (Fig. 6b,c), which was often greatly thinned out and almost undetectable except for intermittent varicosities (Fig. 6b: blue arrowheads). Following cytokinesis, the gradually thickening basal fibre remained associated with one of the daughter cells, while the other daughter cell regrew a separate basal fibre (Fig. 6c).

The thinning of the basal process during cell division precluded us quantifying this feature using timelapse imaging. However, immunostaining against pVIM provided further evidence of RG basal fibre retention, as we observed examples of anaphase and telophase cells exhibiting asymmetric association with the basal process through one of the daughter cells (Fig. 6e). Moreover, a notable fraction of total mitotic cells in VZ-like regions of 10-week cortical organoids (79/282= 28%) exhibited a basal process (Fig. 6d). We were thus able to model the characteristic mitotic behaviour of both NE cells and vRGs in cerebral organoids.

## Discussion

In this study, we present evidence for mitotic behaviour that distinguishes NE cells from RG cells. During mitosis, NE cells retract their basal fibres and round up at the apical surface. This behaviour is consistent with epithelial cells in general that are known to round up and lose basal contact during mitosis^49^. In the neuroepithelium, however, this issue has been controversial. Early studies suggested that NE cells behave in a manner similar to other epithelia^17^,^18^, a conclusion that was supported by electron microscopic observations^23^,^27^. Subsequent studies, however, showed that RG cells retain their contact with the basal surface via the basal fibre during mitosis. This feature of RG cells was consistently observed in several species^8^,^3^,^31^,^46^ and led to the idea that retention of the basal fibre during mitosis is a key feature of all NE progenitor cells. However, most of these studies examined the neuroepithelium during stages of neurogenesis. These observations of mitotic behaviour were therefore made on cells that had transitioned to RG stages and were dividing to generate neurons or other progenitor subtypes.

In this study, we have examined the behaviour of NE cells at an earlier developmental stage when they are highly proliferative. We show that unlike RG cells, mitotic NE cells completely retract their basal processes. The daughter cells generated from these divisions extend a new basal process and re-establish contact with the basal lamina. The presence of growth cones on the tips of the regrowing basal processes suggest that an active pathfinding mechanism may exist to allow these cells to re-achieve basal contact. Such a pathfinding mechanisms would enable the regrowing basal process to navigate the crowded and very dynamic epithelial environment and reach the basement membrane.

The mitotic behaviour of the early NE cells in our study is very similar to the mitotic behaviour (symmetric division at the apical surface and retraction of basal process) reported in short neural precursors (SNPs)^11^. Unlike NE cells, however, the basal processes of non-mitotic SNPs have not been reported to reach the pial surface. Further, both cell types also differ in the type of daughter cells that they generate. SNPs are thought to be neurogenic IPCs^12^,^13^ while the early NE cells undergo proliferative divisions to generate more NE cells and are not observed to generate a differentiated cell type.

Several studies have proposed a role for the retained basal fibre during and after mitosis. These include a need to maintain tissue integrity, retain positional information, provide support and direction for migration of daughter neurons generated by mitosis, and more recently to provide an anchor for outer RG (oRG) progenitors generated from the vRG cells^22^,^50^,^51^,^52^. It has also been suggested that mitotic NE cells retain and divide their basal process before anaphase, and that this may be a means of orienting the mitotic spindle and contractile ring during symmetric division^31^,^51^. These studies stress the importance of retaining the basal fibre during mitosis. Our observations, however, suggest that all early NE cells retract their basal fibres during mitosis. Many of the observations regarding the role of the basal fibre were made on RG cells during neurogenic stages of development. The basal fibres of RG cells create the glial scaffold that is used by migrating neurons^50^,^52^ and therefore may need to remain in place while RG cells are cycling. However, our observations on proliferating NE cells were made before neurogenesis, as we did not observe the expression of neuronal markers at this age. Presumably, at the NE stage, when few or no migrating neurons are present, an extensive basal fibre scaffold is not required. Moreover, as only a subset of NE cells is in M-phase at any given time, only a fraction of NE cells are without a basal process. The basal fibres present on the non-M phase cells may be sufficient to provide support, maintain tissue integrity, and retain positional information.

Another key role attributed to RG basal fibres is that they contribute to the maintenance of the basal membrane (BM) and function to obtain growth factor and other extracellular signals from the extracellular matrix (ECM) via integrin signalling^53^,^54^,^55^. Mice with disruptions in the BM have cortical malformations characterized by misplaced neurons and laminar changes but have normal proliferation patterns. When three different mutations were made (in laminin gamma-1 chain, alpha6-integrin and perlecan) in each of which the basal processes of RG cells were unable to contact the BM, changes were observed in neuronal subtype composition and neuronal positioning but with no effect on proliferation, INM, orientation of cell division, or neurogenesis^55^. As these observations were made in knock-out mice, early NE cells in these animals would also have been unable to contact the BM via the basal fibre. It seems likely, therefore, that the basal fibre plays a limited role, if any, in integrating ECM signals to NE cells.

These observations underscore an important distinction between NE cells and RG cells. NE cells are primarily proliferative. By undergoing symmetric divisions that give rise to other NE cells, these cells serve to increase the number of cells in the progenitor pool. RG cells, on the other hand, serve additional roles in expanding progenitor and neuronal diversity and maintaining a stable scaffold for the migration of the diverse cell types generated during neurogenesis^50^,^56^. This idea is further supported by the presence of the occasional RG cell division occurring in the early neuroepithelium and the increasing numbers of RG divisions in older samples. As NE cells transform into RG cells, their mitotic behaviour changes. The basal process no longer retracts at M phase, Instead the basal fibre tends to thin out as cytoplasmic contents stream to the soma and varicosities appear along its length. Immediately following cytokinesis the basal fibre re-expands.

In spite of their distinct mitotic behaviours, NE cells and RG cells exhibit a very similar molecular profile that make it difficult to separate the cell types from one another^51^. Once a large number of NE cells complete the transition to RG, the neurogenic phase of telencephalic development begins^3^. Our observations suggest that in the developing human cortex, this NE transition may occur as early as 8–10 weeks of gestation. The NE mitosis patterns described here thus provide a dynamic confirmation of a key feature of the radial unit hypothesis^16^ of cortical development in humans.

Our experiments on cerebral organoids generated from iPS cells further confirm and extend our observations. Mitotic retraction of the basal process seen in NE cells in primary tissue is observed in the proliferating cells of organoids as early as 5 weeks after differentiation. This behaviour is a consistent feature of the early neuroepithelium observed in all 5-week-old organoids from three independent lines. At later stages of differentiation, we observed that the mitotic behaviour of the proliferating cells had changed and become predominantly RG-like with most of the dividing cells now retaining their basal processes. This *in vitro* model has two important advantages: first, it provides an independent system to validate observations regarding the mitotic behaviour of NE stem cells. Second, it provides a more easily available system that is better suited to molecular and cellular perturbation assays than primary tissue. Such assays can be used to shed light on the molecular pathways that regulate some of the earliest events in cortical development including mechanisms that govern the transition of NE cells to RG cells. We anticipate that future studies based on organoid models will help us to further understand the molecular mechanisms that regulate the cell cycle of NE stem cells, and the transition of NE cells to RG.

## Methods

### Tissue collection

First trimester human brain tissue was collected from elective pregnancy termination specimens at San Francisco General Hospital. Tissues were collected only with previous patient consent for research and in strict observance of legal and institutional ethical regulations. Tissue collection and research protocols were approved by the Gamete, Embryo and Stem Cell Research Committee (institutional review board) at the University of California, San Francisco.

### Viral transfection and live imaging of organotypic slice cultures

First trimester human tissue was transported to the laboratory within 2 h of collection in artificial cerebrospinal fluid (125 mm NaCl, 2.5 mm KCl, 1 mm MgCl_2_, 2 mm CaCl_2_, 1.25 mm NaH_2_PO_4_, 25 mm NaHCO_3_, 25 mm d-(+)-glucose, bubbled with 95% O_2_/5% CO_2_). Neural tissue samples between GW 8 and GW10 were visually separated into broad regions (spinal cord, mid-/hind- brain and telencephalon; Fig. 1a). Each regional fragment was separately embedded in 3–3.5% low melting point agarose in artificial cerebrospinal fluid and sectioned into 250 μm slices in one of the orthogonal planes to expose the neuroepithelium (Fig. 1b). The resultant organotypic slices were distributed for live imaging, gene expression analysis and fixed for immunofluorescence staining.

For live imaging studies, 250 μm coronal vibratome sections were transferred to Millicell-CM slice culture inserts (Millipore) immersed in cortical slice culture medium (60% BME, 25% Hanks, 10% FBS, 1% N-2, 1% B-27, 1% penicillin, streptomycin and glutamine, all Invitrogen, and 0.66% d-(+)-glucose, Sigma-Aldrich). CMV-GFP adenovirus at a dilution of 1:50–1:500 (Vector Biolabs, 1 × 10^6^ colony forming units) was applied to slices that were then cultured at 37 °C, 5% CO_2_, 8% O_2_ for 12-30 h. For timelapse imaging, cultures were then transferred to an inverted Leica TCS SP5 with an on-stage incubator streaming 5% CO_2_, 8% O_2_, balance N2 into the chamber. Slices were imaged for GFP using a 40 × air objective at 4–6 min intervals for up to 3 days with repositioning of the *z*-stacks every 8–10 h.

### Analysis of divisions

Maximum intensity projections of the collected stacks (5–10 μm step size) were compiled, generated into movies, and analysed using Imaris software. Using Screen Protractor (Iconico), cleavage plane angles were determined by calculating the angle between cytokinesis and the apical surface (Fig. 2b,c). To analyse the morphology of the basal process, single stack and maximum intensity projections of individual mitotic cells were examined before, during and after cytokinesis. The presence or absence of the basal fibre was further verified by examining the *yz* and *xz* confocal projection frames of each dividing cell before during and after mitosis. Imaris software was used to generate 3D surface reconstructions of GFP-labelled cells shown in Fig. 3.

### Gene expression analysis

Gene expression was analysed by qPCR on slices prepared as described above. The slices were frozen in liquid nitrogen and stored at −80°. To isolate RNA, slices were lysed with TRIzol reagent. Following chloroform extraction, aqueous phase containing RNA was mixed with 70% ethanol, transferred onto mini spin column, isolated with RNeasy mini kit (Qiagen), and digested with DNase I (Qiagen). DNA/RNA quality and concentration were assessed using a nanodrop. cDNA was prepared with iScript cDNA synthesis kit (Biorad). qPCR was done with SsoFast EvaGreen Supermix (Biorad). qPCR was performed using the following primers: FoxG1 (Forward: 5′-TCA ACG GCA TCT ACG AGT TC-3′; Reverse: 5′-GGG TCC AGC ATC CAG TAG TT-3′), Pax6 (Forward: 5′-AGT GGG TTT GAA AAG GGA AC-3′; Reverse: 5′-ATT GGT GAT GGC TCA AGT GT-3′); Otx2: (Forward: 5′-TAA GCA ACC GCC TTA CG-3′; Reverse: 5′-GCA CTT AGC TCT TCG ATT-3′) normalized to GAPD (Forward: 5′-GAG TCA ACG GAT TTG GTC GT-3′; Reverse: 5′-TTG ATT TTG GAG GGA TCT CG-3′) and ACTB (Forward: 5′-GGA CTT CGA GCA AGA GAT GG-3′; Reverse: 5′-AGC ACT GTG TTG GCG TAC AG-3′). We calculated amplification efficiency (E) for all the primers above, and ensured that % efficiency was close to 100% (±5%) in order to detect 2-fold differences. Relative ratios were determined using Pfaffl method^57^,^58^, taking into account primer efficiency.

### Immunofluorescence staining and confocal imaging

Upon completion of timelapse imaging, brain slices were fixed in 4% PFA in PBS overnight at 4 °C and then transferred to PBS. Slices were subjected to boiling citrate-based antigen retrieval solution for 20 min and permeabilized and blocked in blocking buffer (PBS plus 0.1% Triton X-100, 10% serum, and 0.2% gelatin) for 1 h. Primary antibodies were diluted in blocking buffer and applied to slices for 36 h at 4 °C. Slices were washed with PBS plus 0.5% Triton X-100 and then incubated in secondary antibodies diluted in blocking buffer for 3–5 h. Images were acquired on a Leica TCS SP5 X laser confocal microscope. Primary and secondary antibodies used: goat anti-SOX2 (Santa Cruz Biotechnology, sc-17320, 1:250), rabbit anti-TBR2 (Abcam, ab23345, 1:100), chicken anti-GFP (Aves Labs, GFP-1020, 1:1,000), rabbit anti-FOXG1 (Abcam, ab18259, 1:250), mouse anti-pVIM (Abcam, ab22651, 1:250), mouse anti-phosphohistoneH3 (Abcam, ab14955, 1:200), AlexaFluor 488, 546, 594, or 647-conjugated donkey anti-goat, -rabbit, -mouse IgG (Invitrogen, 1:500), and AlexaFluor 488 donkey anti-chicken IgY (Jackson ImmunoResearch, 703-545-155, 1:500).

### hIPSC culture

All human iPSC studies were approved by the UCSF Committee on Human Research and the UCSF GESCR (Gamete, Embryo, and Stem Cell Research) Committee. The following wild-type human iPSC lines were used in this study: BJ from a newborn male (ATCC); 1323 from a 48-year-old Caucasian female (Cell Applications; 106-05a); WTc from a 30-year-old Japanese male (Conklin lab, Gladstone). These lines were generated with the episomal Y4 plasmid mixture^59^ and characterized in previous studies^60^,^61^. Established hiPSC lines were cultured in mTeSR1 medium (Stem Cell Technologies, 05850) supplemented with Penicillin/Streptomycin/Gentomycin on Growth Factor-reduced Matrigel (BD, 354263)-coated dishes. Accutase or ReleSR (Stem Cell Technologies, 07920 and 05872, respectively) were used for cell passaging.

### Organoid generation and culture

Organoids were generated according to previously published methods^47^. Briefly, hIPSCs were dissociated to single cells in Accutase and re-aggregated using cortical differentiation medium in lipidure-coated 96-well V-bottom plates at a density of 10,000 cells per well, 100 ml. The cortical differentiation medium (Glasgow-MEM, 20% KSR, 0.1 mM NEAA, 1 mM sodium pyruvate, 0.1 mM b-ME, 100 U ml^−1^ penicillin/streptomycin) was supplemented with Rho Kinase Inhibitor (Y-27632, Tocris 1254, 20 mM, days 0-3), WNT inhibitor (IWR1-e, Cayman Chem 13659, 3 mM, days 0-18) and TGF-b inhibitor (SB431542, Tocris 1614, 5 mM, days 0–18). Media changes were performed on days 3, 6 and then every 2–3 days until day 18. On day 18, the aggregates were transferred to low adhesion six-well plates in DMEM/F12 with Glutamax (Lifetech, 10565-018) medium supplemented with N2 (Lifetech, 17502-048), Lipid Concentrate (Lifetech, 11905-031), Fungizone (2.5 mg ml^−1^), penicillin/streptomycin (100 U ml^−1^) and grown under 40% O_2_ 5% CO_2_ conditions. From day 35, FBS (HyClone, SH30071.03, 10% v/v), Growth Factor-reduced Matrigel (BD Biosciences, 354230, 1% v/v) and heparin (Sigma, H3149, 5 mg ml^−1^) were added to the medium.

### Timelapse imaging and immunohistochemistry of organoids

At 5 and 10 weeks of culture (days 35 and 70, ±2 days, respectively), organoids were collected and processed for live imaging or immunostaining. For live imaging, organoids were imbedded in blocks of 3.5% low melting point Agarose in DMEM. Subsequently, 200-mm vibratome sections were generated and transferred into the organoid culture medium containing CMV-eGFP adenovirus (Vector Biolabs, diluted 1:1,000) and incubated overnight at 37 °C, 8% O_2_, 5% CO_2_. On the following day, organoid slices were transferred and immobilized using Matrigel on glass-bottom dishes (MatTek). Labelled cells were imaged at 15–30 min intervals for up to 5 days using an inverted Leica TCS SP5 with an on-stage environment-controlled chamber, streaming 5% O_2_, 5% CO_2_, balanced N_2_. Maximum intensity projections of the collected stacks were analysed using Imaris software. For immunostaining, organoids were fixed with 4% PFA in PBS for 30 min-1hr, dehydrated in 30% sucrose in PBS overnight and imbedded and frozen at –80 °C in blocks of 30% sucrose/OCT compound (50:50 mixture). 16–20 mm cryosections were subjected to heat/citrate-based antigen retrieval for 20 min and permeabilized and blocked with 10% donkey serum in PBS, 0.1% Triton X-100, 0.2% gelatin. Primary antibody incubations were performed at 4 °C overnight and secondary incubations were performed at room temperature for 1–3 h. After primary and secondary antibody incubations, three 20-min washes in PBS were performed. The following primary antibodies were used: SOX2 (Santa Cruz, sc-17320, 1:100), SOX1 (R&D Systems, AF3369, 1:100), phospho-histone H3 (Abcam, ab10543, 1:200), PAX6 (Covance PRB-278P, 1:200), phospho-Vimentin (MBL, D095-3 (ser82) 1:500 and Abcam, ab22651 (ser55) 1:500), N-Cadherin (BD Biosciences, 610920, 1:200), Pericentrin (Abcam, ab4448, 1:1,000). Secondary antibodies were: AlexaFluor 488, 546, 594, or 647 (all used 1:1,000)-conjugated donkey anti-goat, -rabbit, -rat or -mouse IgG (Invitrogen)

### Data availability

The authors declare that all data supporting the findings of this study are available within the article and its Supplementary Information files, or from the corresponding authors upon reasonable request.

## Additional information

**How to cite this article:** Subramanian, L. *et al*. Dynamic behaviour of human neuroepithelial cells in the developing forebrain. *Nat. Commun.* **8,** 14167 doi: 10.1038/ncomms14167 (2017).

**Publisher's note:** Springer Nature remains neutral with regard to jurisdictional claims in published maps and institutional affiliations.

## Supplementary Material

Supplementary Information Supplementary Figures

Supplementary Movie 1 Dynamic appearance of the early human neuroepithelium. Densely labeled slice of GW 9 human neuroepithelium: At this age, the neuroepithelium is pseudo-stratified and highly proliferative. Individual cells are in a very dynamic state throughout the imaging period, either undergoing interkinetic nuclear migration or mitosis at the apical surface.

Supplementary Movie 2 NE cells undergo proliferative divisions and retract their basal process during mitosis. Video of a proliferative neuroepithelial slice in which we have highlighted 2 NE cells undergoing multiple rounds of cell division over a period of 65 hours (imaged every 6 minutes). Each characteristic spindle shaped parent neuro-epithelial cells translocates its nucleus (yellow arrowhead) to the apical surface and begins to round up. At the same time the basal process begins to shorten (blue arrowheads) and retracts towards the nucleus. At mitosis, only the round nucleus is visible. Following mitosis, each daughter cell (magenta and white arrowheads) independently re-grows its basal process (blue arrowheads) and regains the spindle shaped appearance. The daughter cells in their turn undergo interkinetic nuclear migration and translocate to the apical surface for a second round of mitosis during which the retraction and re-growth of the basal processes can again be observed. Scale bar: 40 microns.

Supplementary Movie 3 Mitotic behavior of early RG cells in the human telencephalon. Video of a proliferative neuroepithelial slice with a mitotic RG cell imaged every 5 minutes. Several NE cells are seen dividing by retracting their basal process in this slice. One dividing cell (magenta arrowheads), however retains its basal process during mitosis. As the cell prepares for mitosis, the nucleus moves closer to the apical surface. At mitosis, the basal process (magenta arrowhead) shows significant thinning and is almost invisible except for the varicosities present along its length but it does not shorten. Following mitosis, the basal process thickens again and is more easily seen. Following mitosis, one daughter cell remains near the apical surface while the other daughter cell can be seen migrating away along the basal process of its sister cell. Scale bar: 30 microns.
