# Supplementary material for: Dynamic behaviour of human neuroepithelial cells in the developing forebrain
Source: Nat Commun. 2017 Jan 31;8:14167. doi: 10.1038/ncomms14167 (PMC5290330; doi:10.1038/ncomms14167)
Supplement: Supplementary Information — Supplementary Figures [file ncomms14167-s1.pdf]

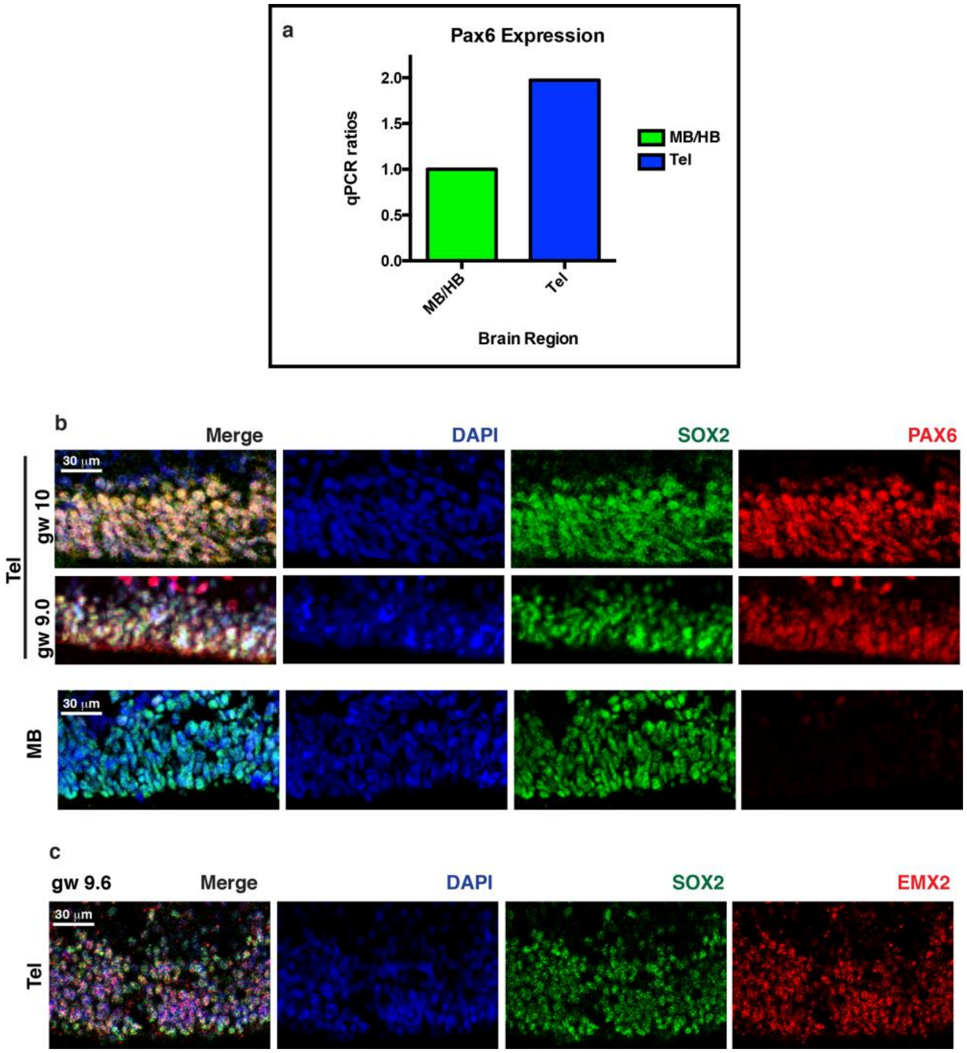

Supplementary Figure 1:

Expression of regional markers in the NE

(a) qPCR analysis of a GW8.4 sample shows a nearly 2-fold increase in the expression of dorsal marker *PAX6* in a telencephalic slice over a mid-brain slice from the same sample.

(b) Immunofluorescence data showing nuclear expression (co-localization with DAPI in single plane confocal images) of PAX6 in the SOX2 expressing cells of the telencephalon whereas SOX2-expressing cells of the mid-brain do not express PAX6

(c) SOX2 expressing cells in the telencephalon also express the dorsal marker EMX2

Scale bars: 30 microns

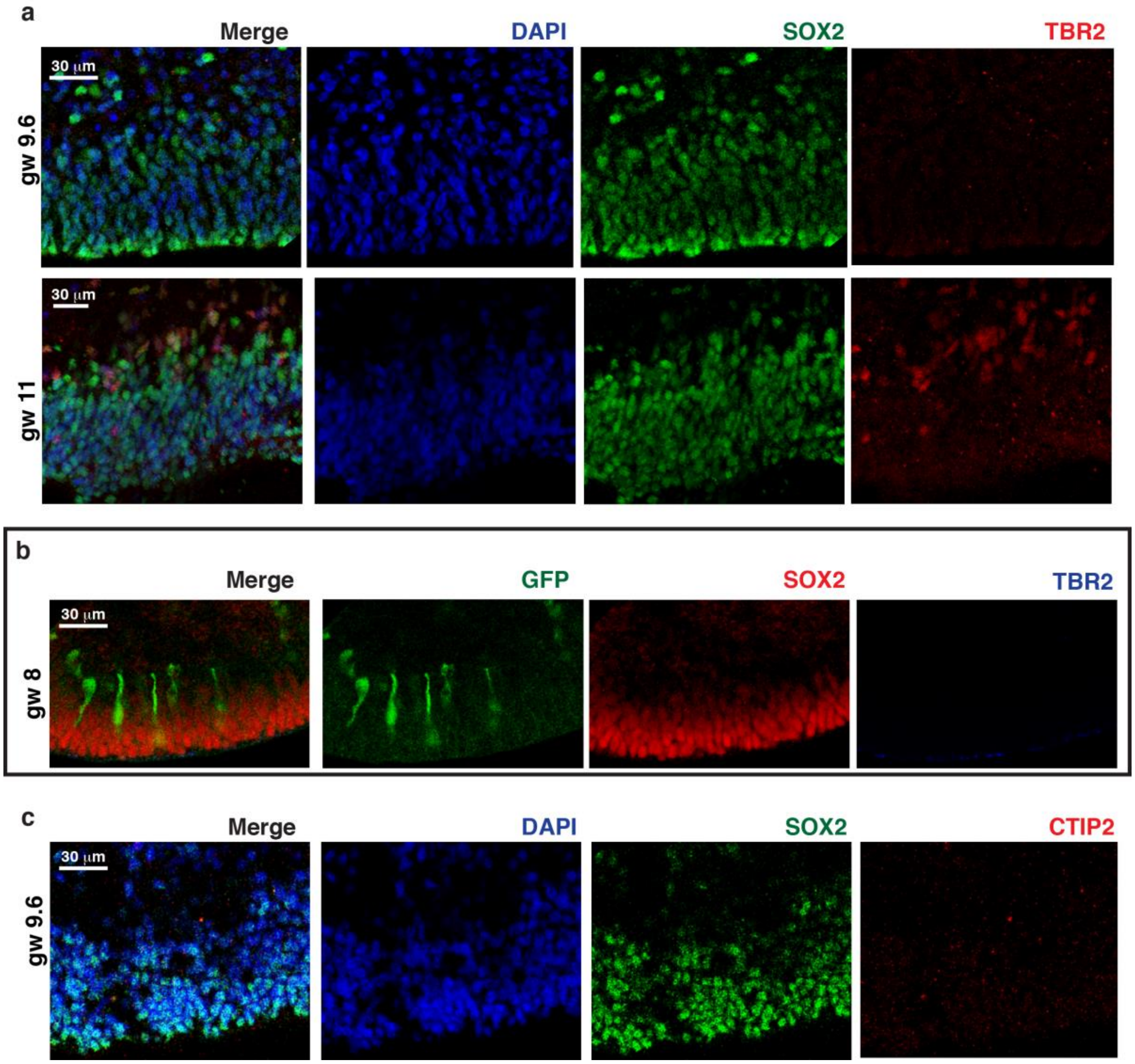

Supplementary Figure 2:

Differentiation in the NE

(a) In early samples (GW8- GW10), the neuroepithelium uniformly expressed SOX2 but not TBR2. By GW11, TBR2 staining can be seen in the nuclei of cells in the intermediate zone (colocalized with DAPI)

(b) No TBR2 expression was seen in GFP-expressing NE cells after live imaging

(c) Neuronal marker CTIP2 was also absent from the early neuroepithelium

Scale bars: 30 microns

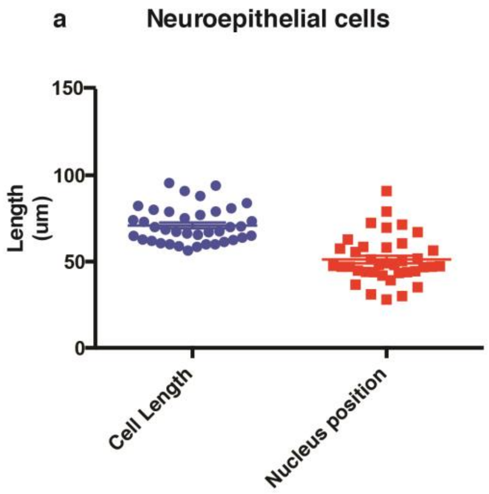

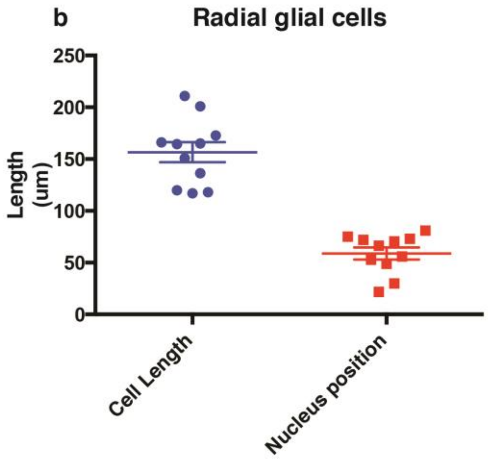

Supplementary Figure 3:

Cell length and inter-kinetic nuclear migration in NE and RG cells in the early human neuroepithelium

Scatter plots showing the length of individual NE and RG cells as well as the position of their nuclei. We used Imaris software to measure cell lengths. The length of the cell was defined as the distance between the point of apical contact of the cell and the tip of its basal process. The nucleus position was the maximum distance traveled by the nucleus along the length of the cell. For this value, we measured the distance

between the point of apical contact of the cell and the farthest position of the nucleus on its basal side during interkinetic nuclear migration using Imaris software.

(a) Scatter plots showing the length of individual NE cells as well as the position of their nuclei. Individual dots represent cell length of each of 38 cells and the position of their nuclei from 3 independent samples. Most of the nuclei travel almost the entire length of the NE cell during inter-kinetic nuclear migration. The mean cell length for NE cells was 70.95±10 um (S.D.) while the mean nucleus position for the NE cells was 51.26±13 um (S.D.). The error bars on the graph represent the S.E.M.

(b) Scatter plots showing the length of individual RG cells as well as the position of their nuclei. Individual dots represent cell length of each of 12 cells and the position of their nuclei from 5 independent samples. The nuclei of RG cells only move a short distance from the apical surface during inter-kinetic nuclear migration. The mean cell length for RG cells was 156.6±32 um (S.D.) while the mean nucleus position for the NE cells was 58.82±19 um (S.D.). The error bars on the graph represent the S.E.M.

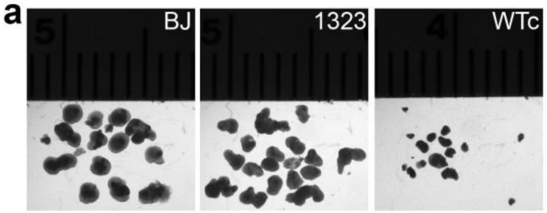

b

Summary of cortical specification	BJ	1323	WTc
Number of experiments that generated PAX6+/CTIP2+ organoids	2	5	1
Total number of differentiation experiments that were performed	6	5	4
Inter-experimental efficiency of cortical neuroectoderm specification	33%	100%	25%

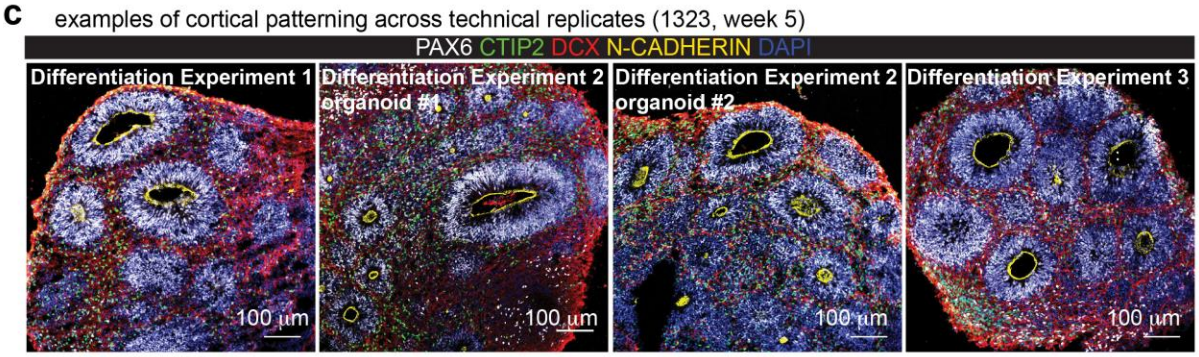

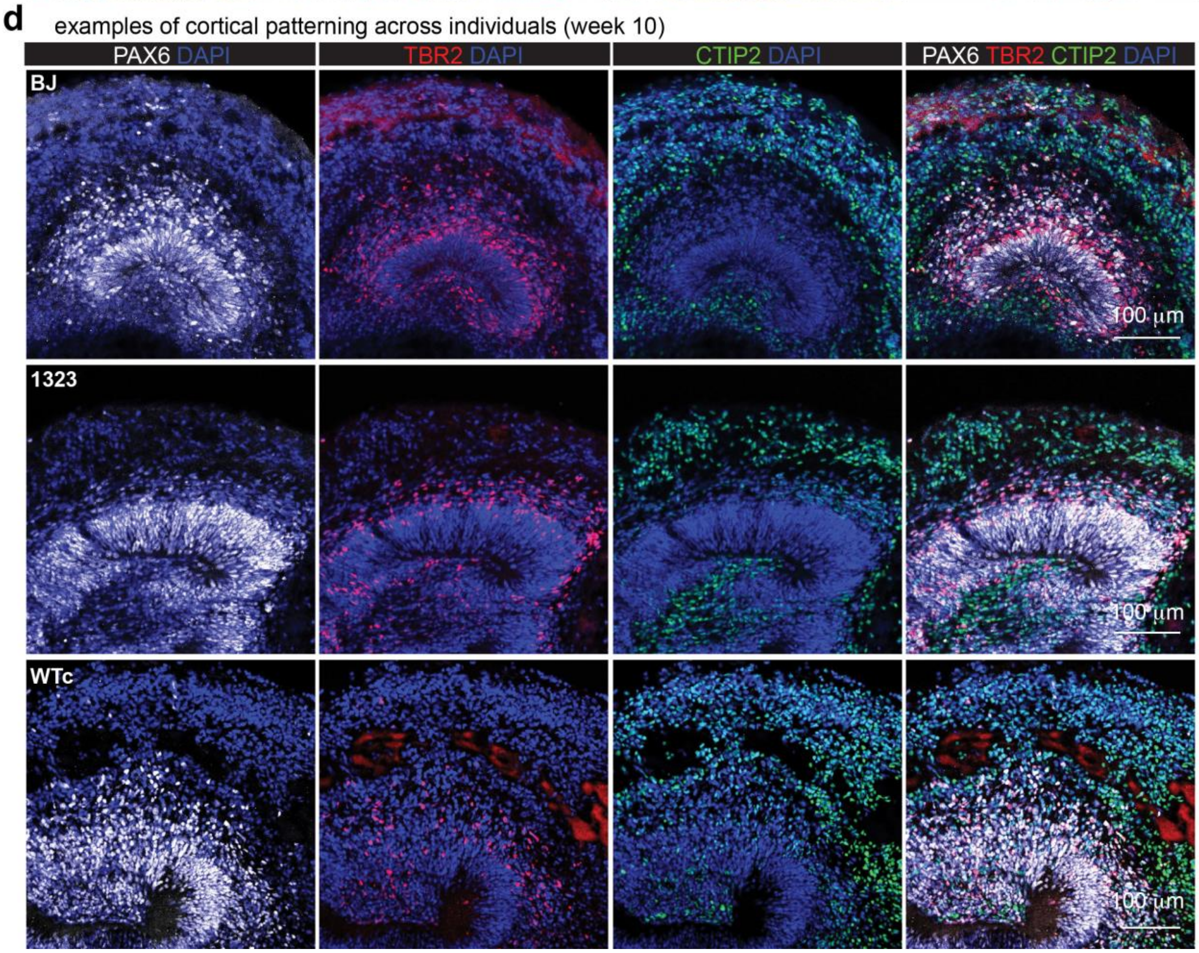

Supplementary Figure 4:

Characterization of cortical patterning in organoids

(a) Cerebral organoids were generated from WT iPSCs derived from 3 healthy individuals. After five or ten weeks of differentiation, 10-20 organoids from each individual were sectioned and stained. This was repeated in 4-6 independent differentiation experiments for each line to determine the efficiency of differentiation (b).

(c) At five weeks the organoids contain multiple pseudo-stratified VZ-like progenitor zones whose apical surfaces are identified by N-CADHERIN expression. Each zone expresses dorsal telencephalic marker PAX6 in a broad VZ-like region, and is surrounded by a very narrow band of DCX-positive neurons that also express the deep layer subcortical projection neuron marker CTIP2. This patterning is consistent between "sister" organoids from a given differentiation experiment.

(d) At ten weeks of differentiation, cortical organoids from all 3 lines begin to express the intermediate progenitor cell (IPC) marker TBR2.

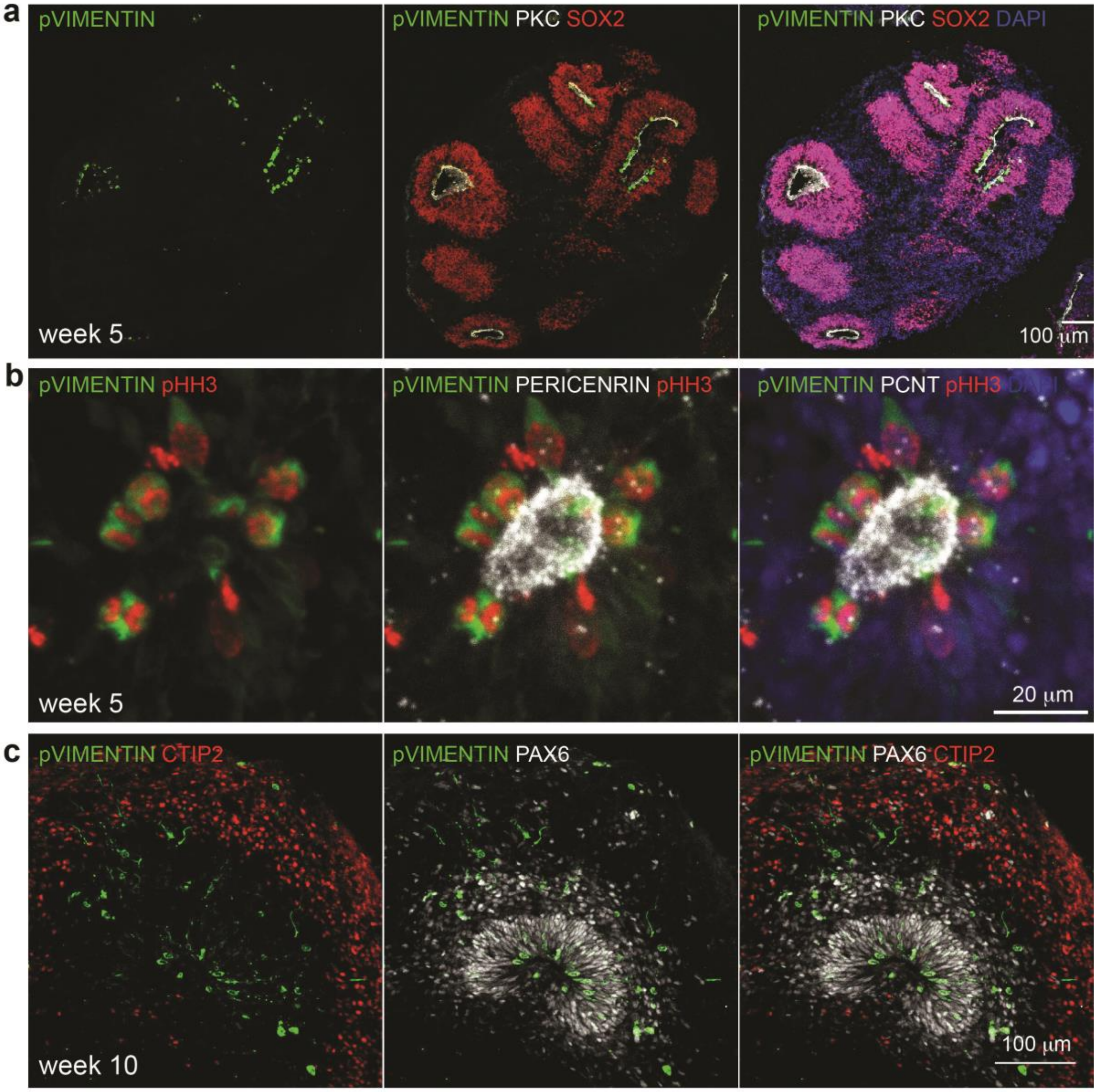

d

Summary of basal process quantification by ICC	5 weeks	10 weeks
Number of iPSC lines analyzed	3	2
Number of independent experiments per line	2	2
Total number of organoids examined	19	14
Number of dividing cells w/ remaining pVIM+ process	31	79
Total number of dividing cells analyzed	365	282
Percent of dividing cells w/ pVIM+ process	8.5	28.0

Supplementary Figure 5:

Characterization of basal process in dividing NE and vRG cells in organoids

(a) At five weeks, pVIMENTIN expressing mitotic cells are located at the apical surface (identified by PKC expression) in cerebral organoids. (b) The mitotic stage in the apical pVIMENTIN-expressing cells is identified by the distribution of chromatin in DAPI staining and pHH3 expression as well as by the position of the centrioles labeled by pericentrin. (c) pVIMENTIN staining highlights the presence of the basal process in several apical mitotic cells in 10 week old organoids.

(d) Summary of actual number of experiments and individual cells examined in live-imaging organoid studies provides evidence of robust basal process retraction in NE cells from week 5 organoids.
